# Sociodemographic determinants of health inequities in low back pain: a narrative review

**DOI:** 10.3389/fpubh.2024.1392074

**Published:** 2024-09-11

**Authors:** Janny Mathieu, Kamille Roy, Marie-Ève Robert, Meriem Akeblersane, Martin Descarreaux, Andrée-Anne Marchand

**Affiliations:** ^1^Department of Anatomy, Université du Québec à Trois-Rivières, Trois-Rivières, QC, Canada; ^2^Faculty of Medecine, Université de Montréal, Montréal, QC, Canada; ^3^School of Medicine, Royal College of Surgeons in Ireland Bahrain, Busaiteen, Bahrain; ^4^Department of Human Kinetics, Université du Québec à Trois-Rivières, Trois-Rivières, QC, Canada; ^5^Department of Chiropractic, Université du Québec à Trois-Rivières, Trois-Rivières, QC, Canada

**Keywords:** low back pain, health equity, access to care, care pathways, sociodemographic characteristics

## Abstract

**Background:**

Health equity is defined as the absence of unjust and avoidable disparities in access to healthcare, quality of care, or health outcomes. The World Health Organization (WHO) has developed a conceptual framework that outlines the main causes of health inequalities and how these contribute to health inequities within a population. Despite the WHO implementing health equity policies to ensure accessibility and quality of healthcare services, disparities persist in the management of patients suffering from low back pain (LBP). The objective of this study was to review the existing evidence on the impact of health inequities on the care trajectories and treatments provided to individuals with LBP.

**Methods:**

A narrative review was performed, which included a literature search without language and study design restrictions in MEDLINE Ovid database, from January 1, 2000, to May 15, 2023. Search terms included free-text words for the key concepts of “low back pain,” “health inequities,” “care pathways,” and “sociodemographic factors.”

**Results:**

Studies have revealed a statistically significant association between the prevalence of consultations for LBP and increasing age. Additionally, a significant association between healthcare utilization and gender was found, revealing that women were more likely to seek medical attention for LBP compared to men. Furthermore, notable disparities related to race and ethnicity were identified, more specifically in opioid prescriptions, spinal surgery recommendations, and access to complementary and alternative medical approaches for LBP. A cross-sectional analysis found that non-Hispanic White individuals with chronic LBP were more likely to be prescribed one or more pharmacological treatments. Lower socioeconomic status and level of education, as well as living in lower-income areas were also found to be associated with greater risks of receiving non-guideline concordant care, including opioid and MRI prescriptions, before undergoing any conservative treatments.

**Conclusion:**

Persistent inequalities related to sociodemographic determinants significantly influence access to care and care pathways of patients suffering from LBP, underscoring the need for additional measures to achieve equitable health outcomes. Efforts are needed to better understand the needs and expectations of patients suffering from LBP and how their individual characteristics may affect their utilization of healthcare services.

## 1 Introduction

Health equity is defined as the absence of unjust and avoidable disparities in access to healthcare, care quality, or health outcomes (1). Health inequalities relate to observed differences in health status or the distribution of health determinants within a population (1–3). To support policy-makers, researchers and practitioners, more than thirty theoretical frameworks on the determinants of health have been developed over the years (4). A review conducted by the Canadian Council on Social Determinants of Health identified the World Health Organization (WHO) Commission on the Social Determinants of Health’s conceptual framework as one of the most comprehensive and relevant to the Canadian context (4). This conceptual framework delineates the fundamental causes of health inequalities within a population (5) and asserts that health inequalities may stem from both the socio-economic and political context of a population (e.g., economic, social, and public policies) and individual-level social determinants (e.g., socio-economic status, gender, ethnic origin, behaviors, biological factors, and living environment) (5). In 2024, the WHO released the “Operational Framework for Monitoring Social Determinants of Health Equity, which builds on previous work led by WHO, other United Nations agencies and stakeholders, and shows the multiple and complex causal pathways through which social determinants of health impact on health equity (6). This framework identifies six domains (i.e., [1] education access and quality, [2] health care and quality, [3] neighborhood and built environment, [4] social community and context, and [5] economic stability), each playing a significant role in health equity (6). Systematic health inequalities are noted when differences consistently appear among groups with distinct socio-demographic characteristics (7). Health inequities, on the other hand, refer to a subset of health inequalities considered as unfair or unjust. The measurement of health inequities considers equity stratification factors, reflecting distinct characteristics for defining and comparing population subgroups (8). The primary equity stratification factors (i.e., age, gender, sex at birth, income, racialized group, education, geographic location, and indigenous identity) identified by The Canadian Institute for Health Information (CIHI) reflect a subset of factors within the conceptual framework of the WHO (1). Health inequities are believed to have an impact on mortality, life expectancy, mental health, and the prevalence of chronic diseases such as arthritis, asthma, diabetes, and obesity (2). Enhanced understanding of health inequalities is crucial for promoting the implementation of collective actions aimed at reorganizing healthcare resources, thereby mitigating or eliminating health inequities.

Low back pain (LBP) is a prevalent symptom affecting all age groups and societies (9, 10). In 2020, 619 million people were affected by LBP globally, and projections indicate an anticipated rise to 843 million individuals over the next three decades (11). Low back pain has become the leading cause of disability worldwide (12), with significant increases observed in low-income and middle-income countries (13). Most cases of LBP are non-specific, characterized by biophysical, psychological, and social dimensions affecting function, societal participation, and financial well-being (14). A closer examination unveils disparities linked to the assessment and management of this condition. In several western countries, the landscape of care for LBP is diverse, encompassing both public and private options, covered or not by individually purchased insurance plans and publicly funded healthcare coverage (15). This provides individuals access to a range of treatments, including publicly funded healthcare service systems, as well as a wide range of private sector services. Based on personal preferences, financial considerations, and the extent of insurance coverage, patients are provided with a range of therapeutic options to manage their LBP (15).

Despite the WHO’s efforts to implement health equity policies aimed to ensure the availability, accessibility, affordability, and quality of prevention strategies, treatments, and healthcare services and programs, challenges persist (16). Even in the most egalitarian societies, individuals with chronic musculoskeletal (MSK) disorders, including patients with LBP, encounter disparities in care, resulting in unequal health outcomes (17). The importance of equity is growing within Canadian healthcare systems, being a fundamental element in evaluating system performance and care quality. By assessing disparities among pertinent population subgroups, we can pinpoint areas for enhancement in healthcare delivery and service utilization, thereby gauging progress towards achieving health equity. In our current understanding, no study has addressed this issue for patients suffering from LBP. Therefore, the study aims to review the existing evidence on the impact of health inequities on the care trajectories and treatments provided to individuals with LBP.

## 2 Methods

A narrative review was performed. This type of review provides a flexible approach in the analysis and interpretation of the literature. A literature search without language and study design restrictions in MEDLINE Ovid database was performed, from January 1, 2000, to May 15, 2023. Search terms included free-text words for the key concepts of “low back pain,” “health inequities,” “care pathways,” and “sociodemographic factors.” The search strategy also included keywords for each equity stratification factor recognized by the CIHI. Reference lists from relevant articles were hand-searched for additional relevant papers.

To be included, studies had to (1) focus on adults (aged >18 years) suffering from any type of LBP with or without radiating pain, (2) investigate at least one sociodemographic determinant of health inequities, and (3) provide data on the impact of sociodemographic determinants on care trajectories or treatments for individuals with LBP. We have also focused our analysis on studies carried out in North America, as the organization of healthcare systems differs between countries, which may impact on patients’ trajectories and the treatments they receive. Study exclusion criteria included: studies focusing solely on the impact of sociodemographic determinants on the prevalence of LBP or on operative and non-operative treatment outcomes (e.g., pain intensity, disability, quality of life), unpublished manuscripts, books and book chapters, conference proceedings, meeting and conference abstracts, thesis and dissertations, and study not reporting on methodology.

Independent reviewers (M.-È.R., M.A.) used a two-phase (titles and abstracts; full-text articles) screening process to select eligible studies. A third reviewer (J.M.) was involved if consensus could not be reached.

## 3 Results

**Figure 1:**
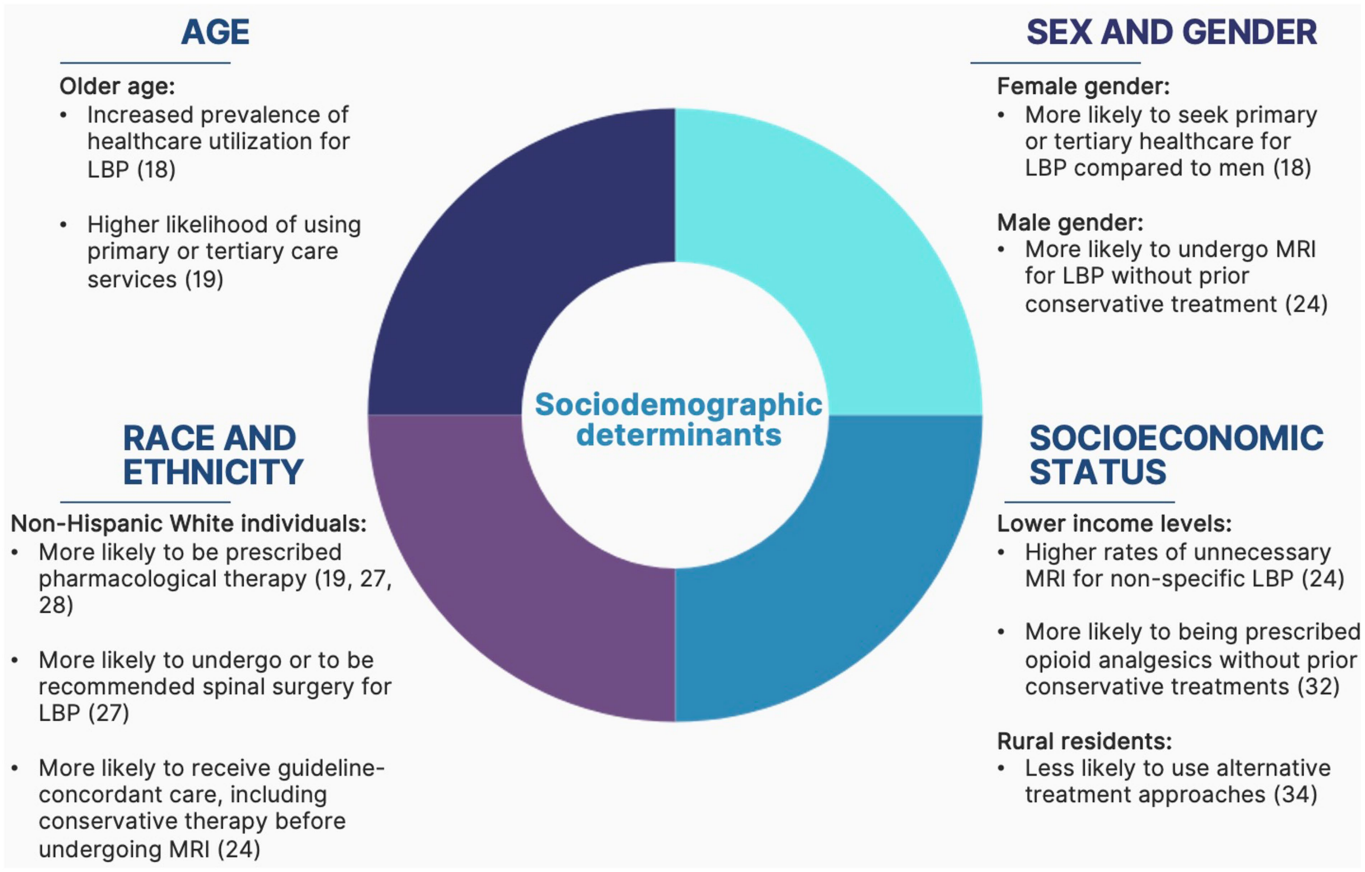
Summary of health inequities associated with sociodemographic determinants in patients with low back pain.

A total of 10, 329 articles were identified from the literature search, which included 10 eligible studies. The evidence regarding the association between sociodemographic health determinants such as age, biological sex, and gender and the care pathways of patients with LBP is limited and considerably heterogeneous. However, some tendencies can be observed regarding the impact of these determinants on healthcare accessibility and utilization for patients with LBP. For each equity stratification factor or group of factors, an overview of health inequities arising from these factors is first described, followed by a description of the inequities observed in the management of patients suffering from LBP. Figure 1 provides a summary of the health inequities associated with each of the stratification factors.

### 3.1 Age

Several organizations, including the WHO (16, 18). The structure and delivery of health care and social services may vary over the lifespan and can notably impact the nature and accessibility of healthcare services (1). For instance, turning 18 defines a transition in health care services from pediatric to adult services (2). This transition notably implies that seeking health care now falls under an individual responsibility (1). Age can positively or negatively impact access to specific healthcare services. In several Canadian provinces, services such as prescription drug coverage and vision care become universally accessible (i.e., are provided or covered by the government or a public health insurance program) from the age of 65. On the other hand, the transition to adulthood may disrupt or reduce access to healthcare services, notably mental health services (19).

Several studies have pointed to age-related differences in the type of care provided and healthcare utilization for LBP. A systematic review analyzing the prevalence rate of health care utilization for LBP, involving nearly 20,000 participants in 11 different countries, found a statistically significant association between the prevalence of consultations for LBP and age (20). Out of 12 studies providing evidence regarding factors associated with healthcare utilization for LBP, three studies reported a positive association between increasing age (i.e., being over 60 years old) and the prevalence of healthcare utilization for LBP. This finding is also supported by a recent cross-sectional study conducted by Allen-Watts et al. (21), which found that the likelihood of using primary or tertiary care services increased by 30.0% for every 10 years increase in age in adults with chronic low back pain (OR = 1.03; 95% Cl [1.005, 1.056]).

### 3.2 Sex assigned at birth and gender

In scientific publications, sex and gender are often used indistinctively, which makes it difficult to assess health inequities associated with either of these sociodemographic factors. Several health inequities are seen between men and women. For instance, in Canada, despite men having a life expectancy at birth that is 4.5 years shorter than women, women spend a greater portion of their lives in poor health condition (22). Furthermore, women are more likely to be misdiagnosed and more often offered ineffective treatment approaches for conditions such as mental health disorders or pulmonary and cardiac conditions (23). The WHO and the CIHI also recognize gender as a distinctive health equity stratifier, defined as the normalized or idealized roles, behaviors, activities, and attributes that a particular society considers appropriate for socially different groups (i.e., generally men, women, and gender diverse individuals) (1, 24). These differences in roles and behaviors may lead to gender inequalities and affect people’s access to and uptake of health services, as well as health outcomes they experience throughout the life-course. For instance, it has been demonstrated that men are more likely to reject healthy beliefs and behaviors and tend to suppress their needs and emotions to correspond to the socially idealized form of masculinity (25).

A systematic review conducted by Beyera et al. (20) revealed a significant association between healthcare utilization for LBP and gender. Four studies consistently reported that females were more likely to seek medical attention for their LBP symptoms than men. The cross-sectional study conducted by Allen-Watts et al. (21) also supported this finding, reporting that women had 2 times greater odds of seeking primary or tertiary healthcare for LBP compared to men (OR = 2.09; 95% CI [1.031, 4.228]), although pain severity did not significantly differ between the two genders. Consistent with previous literature, men seem less likely to seek health care services and tend to engage in more unhealthy habits, such as pain avoidance behaviors (21, 25). A secondary analysis of health claims data from several American hospital centers (26) also revealed disparities in healthcare provided for LBP based on sex and gender. Specifically, this study highlighted that men, compared to women, were 10.0% more likely to undergo magnetic resonance imaging (MRI) for LBP without prior conservative treatment (26).

### 3.3 Race, ethnicity, and cultural identity

Numerous health inequities are related to race (i.e., classification of individuals into groups based on perceived differences in their physical appearance), ethnicity (i.e., belonging to a community or cultural group), and cultural identity (27). Discrimination related to healthcare is said to be indirect when the same services are provided to everyone, appearing equitable. Still, due to cultural, religious, linguistic, or other reasons, some members of minoritized ethnic communities are unable to benefit from them equally (28). Racial inequities are further exacerbated by the combination of racial discrimination and lower socioeconomic status (SES), which are often associated (21).

Race and ethnicity are equity stratifiers known to significantly affect healthcare utilization and care trajectory for patients with LBP. A systematic review conducted by Chen et al. (29) investigated whether there were any ethnicity-related disparities in prescriptions of opioids, advanced imaging, and referrals for spinal surgery among patients with spinal pain. Statistical pooling of 7 studies, all conducted in the United States, revealed that Hispanic/Latinx (OR 0.69, 95% CI [0.49–0.96]) and Black/African Americans (OR 0.59, 95% CI [0.46–0.75]) were less likely to be prescribed opioid analgesics than White individuals. Black/African Americans were also less likely to undergo or to be recommended spinal surgery (OR 0.47; 95% CI [0.33–0.67]) than White individuals. The cross-sectional analysis of Allen-Watts et al. (21) reached similar conclusions, revealing that Non-Hispanic White individuals suffering from chronic LBP were two times more likely to be prescribed one or more pharmacological therapies (OR 2.67; 95%CI [1.23–5.79]) compared to Black individuals. A cross-sectional study of Medicare claim data (i.e., federal health insurance program in the United States that provides coverage for individuals aged 65 and older, and younger individuals with disabilities or specific medical conditions) of primary care encounters also revealed that White patients were more likely to be prescribed opioids for a new onset of LBP compared to Asian, Pacific Islander, and Hispanic patients (30). Differences in access and utilization of complementary and alternative medicine between ethnic groups have also been documented. A secondary analysis using a nationally representative sample of 2009 to 2014 Medicare claim data revealed that non-Hispanic White males (RR 1.10, 95% CI [1.08–1.12]), Black males (RR 1.18, 95% CI [1.10–1.27]), Hispanic females (RR 1.13, 95% CI [1.05–1.22]), Hispanic males (RR 1.24, 95% CI [1.15–1.34]), Asian males (RR 1.13, 95%CI [1.04–1.23]), females of other races (RR 1.24, 95% CI [1.16–1.32]) and males of other races (RR 1.36, 95% CI [1.28–1.46]) were more likely to receive care that was not guideline-concordant, including conservative therapy before undergoing an MRI for non-specific LBP, compared with non-Hispanic White females (26). Although not specific to LBP complaints, a recent scoping review describing chiropractic utilization rate by race, ethnicity, and SES reported that chiropractic utilization was the highest among European American/White/Non-Hispanic White and Caucasian individuals (median 20.00%; IQR: 2.70–64.60%) and the lowest among Hispanic individuals (median 3.90%; IQR 2.90–11.50%) (31). Associations with SES and employment status were also noted, as employed patients from high socioeconomic backgrounds reported a higher rate of chiropractic care utilization than patients from low socioeconomic backgrounds who were unemployed (31).

### 3.4 Socioeconomic status

The SES refers to individuals’ or households’ income, educational level, wealth, and prestige (32). It is one of the major factors affecting patients’ access to healthcare services, as well as their care trajectory. Population surveys held in the two most populated regions of the Quebec province, Montréal, and Montérégie, in 2005 and 2010 revealed disparities in healthcare utilization and experience of care based on SES (33). In this study, Ouimet et al. (33) constructed a composite index, referred to as SES, combining annual crude income adjusted to size of household, perception of economic status, and the number of assets (i.e., car, house, savings). Values of SES ranged from 0 to 10, and were further divided into four quartiles: [1] very low SES (0 to 3.6); [2] low SES (4.6 to 6.4); [3] high SES (7.3 to 8.2), and [4] very high SES (9.1 to 10). In both sample years, the low SES (OR 0.82, 95% CI [0.70–0.98]) and very high SES (OR 0.80; 95% CI [0.66–0.97]) were both associated with less emergency room visits and the very high SES with a lower likelihood of frequent visits to a primary healthcare provider (OR 0.69; 95% CI [0.52–0.90]). The likelihood of affiliation to a family doctor increased concurrently with SES (low SES: OR 1.46, 95% CI [1.21–1.76]; high SES: OR 1.88, 95% CI [1.56–2.29]; very high SES: OR 2.03, 95% CI [1.65–2.51]) (33). These differences, likely representing inequities in access to primary care services, remained stable in the 2005 and 2010 samples, reflecting persistent disparities (33). The relative income (i.e., an individual’s or household’s income compared with that of others in society), the educational attainment (i.e., highest level of schooling achieved), and the geographic location (i.e., living in urban or rural/remote areas) are among the equity stratifiers most commonly used by the CIHI for measuring and reporting socioeconomic-related inequalities in the population (1). As individuals’ SES might be difficult to quantify, the neighborhood SES (nSES) is also used as a comprehensive proxy for reporting social inequalities in a specific geographic area by computing different measures related to wealth and income, such as the median household income and the median value of housing units (34, 35).

Several studies have suggested that socioeconomic factors may contribute to LBP treatment disparities. The secondary analysis of Medicare claims conducted by Lind et al. (26) revealed that areas-level incomes $15,000 to $24,999 were associated with a higher rate of unnecessary MRIs for uncomplicated LBP (i.e., undergoing MRI of the lumbar spine without prior conservative treatments) (RR 1.02; 95%CI [1.003–1.03] for areas with 5% of residents at this income level; RR 1.07, 95% CI [1.01–1.14] for areas with 20% of residents at this income level). The study by Gebauer et al. (34) also revealed that greater neighborhood disadvantage was significantly associated with increased LBP severity and the type of treatment received in the early phase of a new LBP episode. More specifically, patients with low nSES compared with those with high nSES have significantly greater odds of receiving non-guideline concordant care, including favoring the prescription of opioid analgesics over conservative treatments (OR 1.63; 95%CI [1.01–2.62]). A cross-sectional survey of a representative sample of North Carolina residents with chronic LBP also highlighted disparities in the therapeutic approaches used between those living in rural and urban areas (36). In this study, the rural residents questioned were significantly less likely to have used alternative treatment approaches such as spinal manipulation (*p* = 0.01) and spinal traction (*p* = 0.02) or to have sought specialized services for their condition (OR 0.47; 95%CI [0.22–0.99]). An observational study conducted by Bath et al. (37), investigating patterns of healthcare use among adult Canadians with chronic back pain, reached similar conclusions, which were also supported by a recent scoping review exploring chiropractic utilization rates (31), showing that patients with chronic back disorders with lower educational attainment and lower income were more likely to receive a predominantly medical approach to care (fully publicly funded in Canada) and were less inclined to seek non-pharmacological approaches not covered by the government or a public insurance program, such as physiotherapy or chiropractic treatments. Finally, the systematic review by Karran et al. (38) identified one study examining the associations between social determinants of health and care utilization for LBP. This cross-sectional study conducted in North Carolina found that individuals who were insured and had higher educational attainment were more likely to have seen a healthcare provider for LBP in the previous year, but these characteristics were not associated with narcotic use (39).

## 4 Discussion

This study aimed to review the existing evidence on the impact of health inequities on care trajectories and treatments for individuals with LBP and identified several inequities based on CIHI’s inequity stratification factors. This study stands out from previous reviews by offering a comprehensive overview of how sociodemographic determinants impact the care trajectories and treatment options for patients suffering from LBP in North America. The focus on North America in this review offers a unique insight into how sociodemographic determinants affect patient care, minimizing the potential confounding factors related to healthcare system organization that could impact the care utilization and the types of treatments delivered. Such understanding represents an asset for policy-makers and healthcare providers, as it highlights population subgroups around which equitable care strategies should be developed as a priority.

Several studies indicated that older and female individuals were more likely to seek medical attention for LBP. The documented age and sex-related differences in healthcare utilization and care trajectories for LBP are consistent with the overall prevalence of the condition, which is known to be higher among females compared with males across all age groups and to progressively increase with age (10). This finding also reflects the age-related increase in the prevalence of degenerative lumbar conditions, which account for a significant proportion of cases deemed likely to require specialized care services (40). The positive association between age and the utilization of healthcare services could also be explained by insurance coverage. For instance, in the United States, older adults tend to be enrolled in Medicare by age 65 and thus have increased access to health providers compared to younger adults living in low socioeconomic or under-resourced areas (41). Furthermore, it is well documented that older adults seek providers to a greater extent as they tend to experience age-related decline in physical function and are at greater risk for multiple comorbidities (21, 42). The secondary analysis conducted by Lind et al. (26) also revealed that men suffering from LBP were more likely to be directly prescribed further diagnostic testing, such as imaging procedures, before attempting any conservative treatments. Studies suggested this may be explained, though not exclusively, by our social constructs, which are inclined to attach greater importance to the suffering expressed by men (43, 44). In a cross-sectional pilot study, Prego-Jimenez et al. (45) interviewed 80 health professionals and nursing/medicine students and revealed a significant association between the legitimization of LBP and particular beliefs related to sexism and gender roles. More specifically, it has been reported that health professionals tend to view pain in female patients as less believable, less disabling, and less severe. These perceptions may contribute to weakening their inclination to provide support to female patients, especially when no clear pathology is present (43). The stereotype of women being providers and not recipients of care, as well as the belief that women are able to support a greater deal of pain, plays a role in their pain being less validated and less likely to be provided for accurately (44). Men, on the other hand, are thought to require more proactive care from healthcare professionals as their suffering is perceived as more believable (45).

Several studies also suggested that ethnic and racial stereotypes may also contribute to health disparities. A study conducted by Hoffman et al. (46) reported that healthcare professionals’ belief that Black individuals may have a greater biological tolerance to pain could potentially account for the lower prescription of pain-relief medication. Furthermore, it was also shown that individuals from Non-White ethnic communities were less likely to be prescribed opioid medication due to preconceived ideas that they had higher risks of opioid abuse and tend not to comply with medical recommendations (21, 46). Patient-provider racial, cultural, and linguistic discordance were also listed as factors that could contribute to racial inequalities in health care (47). Although not specific to LBP patients, the systematic review conducted by Shen et al. (48) found that racial discordance predicted poorer patient-provider communication, notably affecting information-giving, patients’ satisfaction, and patients-providers’ ability to engage in shared-decision making. Although communication issues may be reflective of race-related attitudes and biases among healthcare providers, studies suggest these may also be explained by patients’ communication effectiveness and a lack of knowledge on how to navigate the healthcare system (26, 34, 36). While questioning Canadian immigrants’ perspectives on healthcare services, Pandley et al. (49) also highlighted that language and ethnic barriers not only impacted timely access and quality of healthcare, but also negatively affected health outcomes by interfering with healthcare providers’ ability to provide health education and recommendations that meet patients’ needs and expectations. In Canada, First Nation people and immigrants who move to urban areas for healthcare face a heightened vulnerability to indirect discrimination, as they encounter difficulties in navigating the healthcare system, accessing services, and following treatment recommendations (50). Engaging in shared decision making is also hindered by the limited interactions of healthcare providers working in large urban centers with individuals from ethnic minorities, undermining trust between patients and providers (48, 49). Studies also suggest that patients’ communication ability may influence consultation outcomes. Indeed, as patients may feel reluctant to ask questions and share health information when they face language, ethnic, or cultural barriers, physicians may, in turn, misinterpret this reticence as passivity, ultimately exacerbating health inequities (48). Minoritized ethnic groups’ attitudes and preferences can also dictate whether to seek health care and the type of treatment a patient turns to. For instance, cultural stigma surrounding mental health and opioid use in the non-Hispanic Black community may explain, though not exclusively, the underutilization of pharmacological therapy for pain relief in patients with chronic LBP (51).

Households’ or individuals’ income, geographic location, and educational attainment were also identified as key factors contributing to disparities in LBP treatments. Lower socioeconomic status and level of education, as well as living in lower-income areas were all associated with greater risks of receiving non-guideline concordant care, including opioid and MRI prescriptions, before undergoing any conservative treatments (20, 21, 26, 31, 34). As a potential explanation for these health disparities, most studies suggest that they reflect differences in insurance status and the variable distribution of uninsured population across regions (20, 34). Individuals with lower SES may face barriers to obtain adequate health coverage, which could hinder access to complementary and alternative medicines and prompt a more immediate reliance on publicly funded care. Beyera et al. (20) also raised the issue of the availability and accessibility of healthcare services, which could explain deviations from evidence-based guidelines for the treatment of LBP. Rural communities seem particularly affected by the lack of accessibility to conservative treatment options. Crockett et al. (52) established that, while approximately 36.0% of the population of Saskatchewan live in rural areas, only 10.0% of physiotherapists practice in these communities (53, 54). Shortage of healthcare providers in these regions emerged as a substantial barrier to access to care and contribute to perpetuating inequities in LBP experiences and outcomes (52). Consistent with these findings, Côté et al. (55) stated that the limited use of chiropractors in rural areas was primarily due to financial barriers associated with traveling to clinics (e.g., taking days off work, cost of transportation) rather than the decision to seek care. Finally, the authors evoke that health literacy (i.e., personal knowledge and competencies that enable people to access, understand, appraise and use information and services in ways that promote and maintain good health and well-being for themselves and those around them) (56), closely related to educational attainment, may also affect the accessibility and the quality of care provided to patients with LBP. It is hypothesized that patients with lower health literacy may struggle to engage in guideline-concordant care due to a lack of understanding regarding recommended medical practices (34).

### 4.1 Strengths and limitations

This review provides a summary of the current evidence investigating the impact of health inequities on care trajectories and treatments for individuals with LBP. To our knowledge, this is the first study to focus on this topic, with a specific emphasis on North America. However, this narrative review used a flexible approach for knowledge synthesis, some limitations. The literature search did not follow an exhaustive systematic search strategy, potentially resulting in relevant studies being missed. Four of the included studies were cross-sectional, which prevents from establishing causal associations between sociodemographic determinants and health inequities. Furthermore, the study populations were often limited to Medicare beneficiaries (adults aged 65 and older), which means that the results from these studies are not generalizable to younger individuals. Additionally, variations in healthcare providers’ specialties were frequently not accounted for, which might have led to inconsistent treatment recommendations and prescriptions. Therefore, in these cases, differences in treatments and care trajectories may reflect clinicians’ experiences and fields of expertise rather than indicating health inequities. Finally, the results of this review must be interpreted with caution, as a comprehensive evaluation of studies ‘quality was not conducted.

### 4.2 Future research directions

The presence of health inequities throughout the care pathways of patients suffering from LBP is a worrying issue and calls for action to develop interventions and programs that will ensure equitable access to guideline-concordant care for all. Future research is needed to develop and evaluate the effectiveness of interventions aimed at mitigating the sociodemographic disparities in healthcare utilization for LBP. This review suggests future research should explore the effectiveness of culturally tailored health education programs and the implementation of patient navigation services to assist individuals from minority and low socioeconomic backgrounds in navigating the healthcare system. Additionally, it seems appropriate that healthcare organizations invest in cultural competence training for providers to reduce biases and improve patient-provider communication. Enhancing availability and accessibility to healthcare services by increasing the pool of publicly funded conservative treatment options and by promoting telehealth initiatives could also contribute to reduce geographic disparities, particularly in rural and underserved areas. Moreover, it appears warranted to explore the appropriateness of implementing policies to improve insurance coverage and affordability of care for lower-income populations, ensuring that financial barriers do not hinder access to evidence-based treatments. Finally, this review suggests that public health initiatives could also benefit from focusing on increasing patients’ health literacy, particularly in communities with lower educational attainment, to empower patients with the knowledge and skills needed to engage in guideline-concordant care.

## 5 Conclusion

Although LBP affects individuals of all sociodemographic backgrounds, studies have revealed that sociodemographic determinants such as age, sex, gender, race, ethnicity, and SES can shape care pathways of patients suffering from this condition, therefore deepening inequities in healthcare accessibility and quality within a population. While these findings may indicate a need to assist healthcare professionals in promoting just and equitable care delivery, efforts are also needed to understand better the needs and expectations of patients suffering from LBP and how their individual characteristics may affect their utilization of healthcare services.
